# Jejunal Windsock Deformity: A Rare Cause of Incomplete Neonatal Intestinal Obstruction

**DOI:** 10.21699/jns.v5i4.384

**Published:** 2016-10-10

**Authors:** Vijai Datta Upadhyaya, Basant Kumar, Amrit Gupta, Kirti Naranje, Anita Singh

**Affiliations:** 1 Department of Pediatric Surgery, SGPGIMS, Lucknow India; 2 Department of MRH, SGPGIMS, Lucknow India; 3 Department of Neonatology, SGPGIMS, Lucknow India

**Keywords:** Windsock deformity, Jejunal web, Intestinal obstruction

## Abstract

Incomplete intestinal obstruction due to windsock web of the jejunum is uncommonly noticed in neonates. We present a male neonate, prenatally suspected case of proximal bowel obstruction, who was found to have features of incomplete intestinal obstruction due to windsock deformity in jejunum. The difficulty in the diagnosis and management is discussed along with relevant literature review.

## CASE REPORT

A preterm male child, prenatally suspected as a case of proximal bowel atresia on antenatal scan, delivered by caesarian section at 34 week of gestation weighting 2100 gm was referred to our department. The patient passed meconium on day one. Local examination showed fullness in upper abdomen which decreased after nasogastric drainage. Plain X-ray of abdomen showed dilated stomach shadow with decent amount of gas in distal bowel. Ultrasound of abdomen showed bilateral hydroureteronephrosis, distended urinary bladder without dilatation of posterior urethra and mildly dilated stomach. In view of bilious aspirate and prenatal ultrasound finding, upper gastrointestinal contrast study was performed to assess the level of obstruction. Study showed, grossly dilated stomach, all parts of duodenum were dilated with an abrupt narrowing in the proximal jejunum (Fig.1A). On exploration, stomach was grossly dilated and all parts of duodenum were significantly dilated along with proximal 15 cm of jejunum (Fig. 1B). Distal bowel was apparently normal. Transition area showed web with central hole (Fig.1C). Jejuno-jejunostomy was done after excision of the web (wind-sock deformity- WD).

**Figure:**
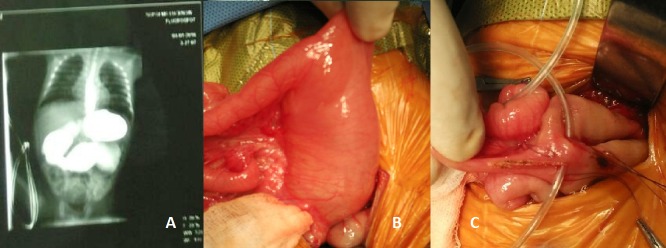
Figure 1: (A) Contrast study showing hugely dilated stomach, duodenum, and proximal jejunum; (B) Peroperative findings of jejunal web; (C) Wind-sock web with central aperture.

## DISCUSSION

Windsock deformity is a very rare with incidence of 1 in 10000 to 1 in 40000 [1]. WD is usually described in duodenum and most commonly seen in second part of the duodenum, it accounts for almost 85 to 90% cases of WD [1]. Most of the rest WD had been described in third or fourth part of duodenum [2]. In present case WD was seen in jejunum which is very rare and less than 10 cases had been reported till now in the English literatures [2-6]. Diagnosis in this type of deformity is mostly delayed and in only two cases it was diagnosed in neonatal period (at or before age of 72 hours). In this index case, proximal bowel atresia was suspected prenatally, but patient passed adequate amount of meconium on day of birth without any anal stimulation and post natal X-ray abdomen showed adequate amount of gas in distal bowel the making the diagnosis of bowel atresia doubtful. Tans-anastomotic feeding for jejuna atresia had not been described though it is used commonly in cases of for duodenal atresia at our centre [7]. Feeding jejunostomy helped in starting early feeding (within 72 hours of surgery). Endoscopic treatment for partial intestinal obstruction such as stenosis and windsock deformity has been described for duodenal web with central hole [1,2] but it was not possible in this patient because of two reasons, one is the patients was only two days old and secondly the deformity was present in proximal jejunum. 

In conclusion, WD of small bowel should be kept in differential diagnoses in cases of neonate presenting with partial intestinal obstruction.

## Footnotes

**Source of Support:** Nil

**Conflict of Interest:** Nil
